# The facilitators of communication with people with dementia in a care setting: an interview study with healthcare workers

**DOI:** 10.1093/ageing/afv161

**Published:** 2016-01-13

**Authors:** Miriam Ruth Stanyon, Amanda Griffiths, Shirley A. Thomas, Adam Lee Gordon

**Affiliations:** 1 Division of Psychiatry and Applied Psychology, School of Medicine, University of Nottingham, Nottingham, UK; 2 Division of Rehabilitation and Ageing, University of Nottingham, Medical School Queens Medical Centre, Nottingham NG7 2UH, UK

**Keywords:** dementia, communication, thematic analysis, care workers, care homes, older people

## Abstract

**Objectives:** to describe the views of healthcare workers on the facilitators of communication with people with dementia in a care setting.

**Design:** thematic analysis of semi-structured interviews.

**Setting:** all participants were interviewed in their place of work.

**Participants:** sixteen healthcare workers whose daily work involves interacting with people with dementia.

**Results:** four overarching categories of themes were identified from the interviews that impact on communication: the attributes of a care worker, communication strategies used, organisational factors and the physical characteristics of the care environment.

**Conclusion:** many strategies used by healthcare workers to facilitate communication have not yet been studied in the research literature. Participants' views on training should be incorporated into future dementia training programmes.

## Introduction

Communication impairments are among the earliest symptoms of dementia. Difficulties range from word finding and comprehension problems to the negative effects of disorientation and anxiety.

Approximately a third of people with dementia (PwD) in the UK live in care homes [1], where effective communication between care workers and PwD has been shown to influence behaviour [2] and well-being [3] in care recipients and burnout rates in care workers [4].

The importance of communication between healthcare workers and those they care for was first highlighted in Kitwood's ‘person-centred care approach’ [5]. Kitwood postulates that an interaction is composed of one person making an action, another responding to that action and the first person then reflecting on that response. When communicating with a PwD, this process becomes even more complex due to inhibited abilities to state intentions, make rational assumptions about the conversation partner and possibly an altered sentient state. Therefore, Kitwood states, communication with a PwD requires considerable resources from the conversation partner; he likens the process to a tennis coach maintaining a rally with a novice.

There is a growing body of literature regarding training healthcare workers in person-centred care [6], but these courses do not focus on specific practical communication techniques. A body of literature on communication between healthcare workers and PwD has suggested speech characteristics such as ‘elderspeak’ [7–10] or a controlling vocal tone [11], cause resistance to essential care activities. Training healthcare workers in communication techniques, such as using short sentences, can reduce depression and increase communicative behaviour in PwD [12–21]. However, current training programmes are based predominantly upon clinical experience, and little work has been undertaken to manipulate communication techniques experimentally to establish empirical gold-standard models of communication.

As a precursor to work using experimental models to investigate the impact of communication techniques on well-being and resistance to care in PwD, we set out to describe how healthcare workers facilitate what they perceive to be effective communication and to learn from the experientially derived body of knowledge held by healthcare workers.

## Methods

### Setting and participants

Participants were healthcare workers currently or previously working with PwD on a daily basis. All participants had done such work for 6 months or more and were English speaking. Eight were care workers from two residential care homes in the Midlands of the UK. Both homes specialised in dementia and had received the highest local authority quality rating. One had 32 beds and one 52 beds. Care workers were accessed via their managers. The other eight participants were healthcare professionals working with PwD on a daily basis. The sampling began with individuals from dementia outreach and care home specialist nursing teams and snowball sampling was used to contact other healthcare workers with experience in dementia. All participants were invited personally by the first author and their voluntary consent obtained.

### Procedures

An interview guide was developed with the aim of constructing a comprehensive picture of the factors impacting on effective communication with PwD. Questions were based on factors identified in the literature referring to speech, challenging behaviour and staff training. The guide was piloted with two experienced healthcare professionals, one therapy assistant and one retired nurse. Pilot data were used to clarify the meaning of questions in the interview guide but were not included in the final analysis. The final guide included questions on: challenges faced when communicating with PwD; facilitators and barriers to successful communication; dementia training and the impact of organisational structures on communication (see Supplementary data, Appendix A, available in *Age and Ageing* online). Semi-structured interviews were conducted in a private room at the participants' place of work and lasted 15–60 (median 34) min. Interviews were audio-recorded and anonymised on transcription. Recruitment ceased when no new topics were generated, and data saturation was thought to have been achieved [22, 23].

### Data analysis

Two coders analysed the transcripts using the six-phase thematic analysis process of Braun and Clarke [22]. Familiarisation with the data occurred through repeated listening to the interview recordings during transcription and repeated reading of the transcripts. NVivo9 was used to store, code and analyse data. Initial coding was extensive and inclusive and later refined to a parsimonious scheme with overarching categories and sub-themes. The internal homogeneity of the themes was largely managed by the first author. The external homogeneity was a discursive process involving the first author, two occupational health researchers and an auditor (second author) who advised and questioned the definition and purpose of themes and sub-themes. The process of refining the themes was thoroughly documented with several versions of the coding scheme developed before deciding on a ‘best fit’ for the data. Themes were generated inductively from a realist paradigm [24]. There was no pre-existing framework for coding of themes before analysis.

Ethical approval was granted by the Ethics Committee of the Institute of Work, Health and Organisations, University of Nottingham, UK. This work was supported by the UK's Economics and Social Research Council (studentship award number ES/I021132/1).

## Results

### Study participants

Eight care workers were recruited, five from care Home 1 and three from care Home 2. One mental health nurse, one activities coordinator, two occupational therapists, one clinical psychologist, one speech and language therapist and two consultant geriatricians were also recruited. This later group of eight participants is henceforth referred to as healthcare professionals. Hereafter, when referring to both types of participant (care workers and healthcare professionals) the term ‘healthcare workers’ will be used. All care workers and 8 of 10 healthcare professionals invited to participate did so. Healthcare professionals had a median (range) of 14(3–53) years and care workers 1(0.5–7) years' experience. Seven care workers and three healthcare professionals were female. The healthcare professionals generally worked with their patients within the care home setting and over periods of time in excess of 6 months.

### Findings

Four overarching categories representing factors considered to facilitate communication with PwD were identified: attributes of a care worker; the practical verbal and non-verbal facilitators; organisational factors of home ethos, leadership and training; and the physical environment. For the most part, care workers and healthcare professionals, irrespective of professional background, identified similar issues. Though no definition of communication was given by the interviewer, all participants assumed a broad definition of communication including non-verbal behaviour such as signs of agitation.

#### Theme 1: Knowledge, skills and characteristics of a care worker to facilitate communication

##### Knowledge

All participants considered that a comprehensive understanding of dementia was essential to successful communication. This involved having realistic expectations of what might be achieved through an interaction and what a PwD would be able to understand so as not to burden them unnecessarily. Modifying expectations of how normal conversation should operate included, for example, finding ways to show interest other than using conventional questions that required factual answers.

If you had a picture of a guard from the Tower of London and I said to you “Who is he? What is he?” … generally, people can feel tested … But if you were just generally curious with that person, “Wow that's a really strange hat he's wearing isn't it?”, then people, from a position of strength, can volunteer their own opinions. (Consultant Clinical Psychologist)

Participants spoke about the importance of accepting and accommodating for cognitive deficits during an interaction with PwD; for example, accepting that repetition of instructions or answers to questions is necessary. Most participants spoke of the importance of knowing what they described as the ‘general effects’ of old age on communication abilities, as well as the influence of co-morbid conditions including depression and stroke.

I think what is very difficult working with older people is just the volume and the intensity of the difficulties. Every other patient is deaf, every fifth patient can't see very well, every second patient is immobile … one in seven are distractible. (Consultant Geriatrician 1)

A majority of participants thought it important to regard challenging behaviour, such as agitation or aggression, as attempted communication of unmet need. One participant spoke of the effect of ignoring a person's deficits in an interaction:

…on the one hand we assume that they're incompetent and on the other hand…we argue with them as if they are competent. And then we're very confused, hurt and annoyed when it doesn't help. (Occupational Therapist)

##### Skills

Principally, skills were used with the goal of building relationships with PwD. These relationships were thought to facilitate successful communication. All participants described the need to consider each person as an individual, seeking to know their personality, preferences and abilities. Information could be gathered from PwD's notes, family members and their own and others' experiences with that person. For most participants, these relationships were formed over many months and sometimes years. For the one participant (a consultant geriatrician) who had contact with their patients over a shorter period, it was these skills he described as enabling him to get to know a patient. The importance of reading patients' notes and speaking with other staff members about communication successes or failures with them was emphasised.

You have to look at the patient and know something about their background … and that can give you a real insight into who they are and then you can develop your communication skills for that individual. (Care Worker 1)

All except one participant related how a healthcare worker should have the skills to use every opportunity for contact with a PwD to build relationships. They explained that most interactions with a PwD took place during daily care activities; these were one-to-one opportunities with few distractions for both conversational partners. They also spoke of the importance of purposefully making additional opportunities for communication. Some called this ‘embedded communication’, which mitigated against task-oriented care which they felt tended to prioritise physical over social and emotional needs.

Participants described the importance of care workers showing respect for individual personhood through communication. This could be achieved through politeness and ‘good manners’ but also by giving PwD choices about care and avoiding exposing their disabilities through conversation.

That's a person you have in front of you. No matter what stage of life they're in, acknowledge that they're still a person and talk to them. Communicating is the most important thing in life. (Mental Health Nurse)

Approximately half of the participants thought it imperative to be able to engage with the subjective reality of the PwD by letting go of facts and addressing the emotional reality behind their attempts at communication. This required empathy and a combination of validation and reality orientation determined by the context and level of distress shown by the PwD.

The interaction feels like being in a Beckett play … There is something about being able to tolerate the absurd … They're often quite weird conversations but I think they can still be quite meaningful conversations in an emotional and psychological sense. (Occupational Therapist 1)

##### Characteristics of the care worker

Another set of attributes believed to facilitate communication were related to the character of the care worker. These were often considered as ‘innate’ abilities, nurtured by the care worker through experience.

Patience and tolerance were said to be crucial in coping with challenging behaviour, for example in not taking acts of aggression personally.

You just think, ‘oh she should be behaving that way’ but you just have to go with the flow in a way and try to understand. That's the important bit. Be patient. (Care Worker 2)

Honesty was a controversial topic. Some saw absolute honesty as the moral ideal, but all participants spoke about the importance of being cautious and selective in the elements of truth disclosed to the PwD. This was often referred to as ‘tact’. Participants did not seem comfortable with deception but emphasised that it sometimes became acceptable in the context of preventing unnecessary distress for PwD. Sensitivity to a PwD's responses and mood by attending to often subtle body language were described by the majority of participants as especially important in detecting non-verbal expressions of pain or anger.

It's less about what the [PwD's] abilities are and more about the people around that person, what their abilities are, to enable them to effectively process what's trying to be communicated. (Consultant Clinical Psychologist)

#### Theme 2: Communication strategies

##### Verbal

Participants described many practical ways in which communication with PwD could be facilitated. All participants spoke of reducing speech complexity, making sentences shorter and avoiding the use of abstract ideas.

You might give an instruction like, ‘Stand up’ instead of, ‘Hello John it's really nice to see you. Isn't the weather lovely today? Do you fancy having a stand up and going for a walk with me?’ (Occupational Therapist 2)

Yet it was also noted that oversimplification could lead to infantilisation.

Keeping speech quiet and calm was thought to reduce agitation and confusion. Participants described the negative impact of using a ‘special voice’, sometimes known as altered vocal tone. It was noted by some participants that the ability of PwD to interpret emotional tone is maintained well into the condition.

Special voices … you certainly shouldn't speak to them as though they're a child … they're adults, irrespective of whether they've got dementia … You don't start standing there and saying “Right, this is what's going to happen.” And saying it in a special voice is absolutely drastic. (Activities Co-ordinator)

Participants stated the importance of explaining activities as they took place, repeating instructions, often accompanied by rephrasing; they also reported using simple questions to establish the meaning behind interactions initiated by PwD.

You get an inkling of what they're trying to say, but you have to have a start place ‘Is it in the room?’ and then sometimes they go ‘Yeah’ and when it comes to it it's actually a car outside. But you have to start somewhere haven't you? (Care Worker 4)

##### Non-verbal

Various non-verbal facilitators of effective communication used by healthcare workers included eye contact to communicate emotion and prevent PwD withdrawing from interaction, positive facial expression to communicate openness, touch to initiate and maintain attention, or communication aids such as pictures and gestures to elaborate on verbal communication. Descriptions of non-verbal facilitation were often caveated with reminders that each individual PwD is different and tolerates different levels of eye contact or touch. Such individual preferences could only be learned through a deepening relationship with each PwD. The participant who generally worked with patients over short periods shared the most anecdotes about communication failure due to lack of knowledge about the preferences of an individual. One participant spoke of communication as an intuitive process:

I think non-verbal communication is massive … We do a lot intuitively in terms of, “Is this person somebody I'll get on with? Would I like to have a cup of tea with this person?” … and I don't think that gets lost with dementia. (Speech and Language Therapist)

##### Pacing, disengagement and distraction

Participants stated the importance of pacing activity at a suitable speed. This combined slowing speech with slowing the task so that PwD could keep up both physically and mentally.

I've seen people literally saying “Would you like a cup of tea? Cup of tea? No, ok fine” and away they go and then you hear the person say, “Yes” but by that time they're gone. (Consultant Clinical Psychologist)

A few participants spoke of disengaging from interactions if it was becoming stressful for PwD and sometimes asking another worker to take their place. Nearly half of the participants referred to the use of distraction or rewards when completing a task disagreeable to the PwD, such as cleaning up after an episode of incontinence.

We had a woman here who used to smoke like hell and I used to say “Oh you've had a fall of soot!” And she would laugh and the tension would be gone and … in the time it would take her to stop laughing, it would all be cleaned up and done. (Care Worker 4)

#### Theme 3: Organisational factors that influence communication

##### Culture, leadership and management

All participants commented on how the culture of a care environment influenced their ability to communicate effectively. Most said that effective communication should be encouraged and modelled by managers who make it a priority over other daily tasks. Some spoke of being encouraged by their managers to sit and chat with residents and being criticised for not doing this. Staffing levels were thought to affect the quality of communication, as conversation was perceived to be easily deprioritised when other tasks were pressing.

They [care workers] would love to be able to comfort that lady [PwD] … Part of it will be that they don't know how to respond to her, or that they don't have the numbers to respond with sufficient intensity, or that the leadership is making them tidy the linen cupboard rather than sit with a distressed lady. (Consultant Geriatrician 2)

##### Staff training

Participants suggested that care workers needed better quality training. There was a general consensus as to how current care worker training could be improved. Improvements involved: (i) Being more practical in learning methods and application, (ii) appreciating the existing expertise of the care workers; (iii) Supporting the transfer of training into practice; (iv) The attendance of senior care home managers which, they believed, would facilitate the subsequent implementation in the workplace; (v) Covering social and psychological aspects of care work (for example, empathy) so as to encourage emotionally engagement (in addition to the more conventional skills-based components of training). When referring to a dementia training video filmed from the viewpoint of a PwD, one care worker said:

My heart broke and I thought ‘That ]PwD] could be me!’ And I think that's the thing. It's getting people to think about what it's like from their point of view. (Care Worker 7)

#### Theme 4: Physical characteristics of the home

Finally, participants noted the importance of adequate space and an ambient environment where auditory and visual stimulation were at a suitable level so that PwD were not overloaded. This was thought to help them concentrate on an interaction and prevent them from withdrawing.

Again we've had individuals who are clearly distressed in very noisy, very chaotic environments. So the common sense approach is to put them in a very quiet, calm environment. But that is often as aversive for those individuals as the chaotic environment. (Consultant Clinical Psychologist)

Some of the care workers spoke of a recent extension to their care home's garden that provided a safe environment for PwD to wander outside. This space was thought to allow them a greater sense of freedom, burn off pent-up energy and frustration, and allow them to be more focussed when interacting with care workers.

I've often found that, if they're all pent up and stressed, after a couple of laps, they're much more listening to what you're trying to say. (Care Worker 5)

## Discussion

### Main findings

This study describes the facilitators to communication with PwD as described by experienced healthcare staff providing care on a daily basis. Four main categories of themes that facilitated communication were identified: attributes of a care worker; the practical verbal and non-verbal facilitators; organisational factors of home ethos, leadership and training; and the physical environment.

### Context of previous literature

This study found that personal attributes of care workers were rated as significant factors in communication with PwD. Few prior studies have explored the role of personal attributes in this context. Studies have sought to teach care workers' communication skills [12, 15, 16] or to train them in the skills of sensitivity to non-verbal emotional cues [14] but have not defined this sensitivity as a personal attribute.

Although personhood is a widely used concept in dementia care, the practicalities of endorsing personhood through day-to-day communication are rarely mentioned in previous research. Data presented in this study suggest precise mechanisms by which healthcare workers believe this can be enacted. They describe attempting interaction despite communication difficulties and expressing a wish to develop a relationship with the PwD. They consider the use of good manners and respectful address, and offering of choice to assist the PwD to maintain a sense of agency. These interventions were thought to affirm the PwD's personhood and empower them to continue in communication.

The ways in which characteristics of care workers affect interactions with PwD have not been studied in existing literature. Particular personality traits, such as being slower tempered, more stoic and reflective, have been described previously as more prevalent in the care workforce [25], but the impact of these on communication in care has not been explored. Attributes highlighted in this study could be subjects of future research. These include an attitude of respect, patience and the ability to engage with the subjective reality of another.

The impact of culture and leadership in a care setting has been described in the literature as influencing the quality of dementia care. Leadership can influence the degree to which care workers feel able to interact with PwD [17, 26]. One study found that the culture of a ward did not change a staff member's priorities but could impact their perceived ability to fulfil them [27]. Such findings support the findings in this study and raises care home culture as an important potential confounding variable that could influence communication and any attempts at experimental manipulation.

The two care homes involved in this study used programmes of training administered by different training companies and the healthcare professionals came from different backgrounds with diverse training experiences. However, all participants, irrespective of background, recommended the same improvements to communication training. The observation that dementia training does not adequately focus on the relational elements of care is supported by a published review of care worker training [28]. None of the staff training interventions published in the literature to date consider the emotional or relational element of care work [12–16] and only one involved a process helping students to translate their training into daily practice [29].

### Strengths and limitations

The main strength of this study was the use of semi-structured interviews that allowed healthcare workers to describe in depth the techniques they advocated as enhancing communication with PwD, their rationale for using those techniques and other factors believed to be influential in promoting effective communication. There was strong consensus between participants, albeit from varied professional backgrounds. Its limitations include a reliance on accounts from a small sample. It is possible that a different method of sampling may have resulted in a less homogenous view of the topic. It is not known whether the factors reported as important by participants are used by participants or have an impact on successful communication with PwD. The opinions expressed may not be representative of all those who work with PwD. PwD were not interviewed in this study due to the severity of dementia generally found in care contexts. The opinions of PwD in the early stages of dementia on what facilitates communication with their carers would enrich the discourse on this topic.

### Implications

Participants described new communication techniques not previously noted in the literature. Many of the techniques recommended by participants, such as distraction, disengagement, explanation of actions, pacing and the use of positive facial expression, have not yet been investigated empirically. This might be addressed in future studies. Future studies should also seek to establish the effects of care workers' knowledge, skills and characteristics on successful communication. The results of such studies would inform the design and content of training courses for care workers. Training courses should encourage empathy with PwD, be attended by care staff of all levels of seniority and facilitate the transfer of training into practice.

**Key points**

- Those providing care for people with dementia believe that the personal attributes of the care giver can facilitate communication.
- Healthcare workers describe practical verbal and non-verbal facilitators to communication with people with dementia.
- Organisational factors such as home ethos, leadership and training influence communication.
- The physical environment has an influence on communication.
- There are implications for the development of training resources for care workers.

## Supplementary data

Supplementary data mentioned in the text are available to subscribers in *Age and Ageing* online.

## Conflicts of interest

None declared.

## Funding

M.R.S. was funded by the UK Economics and Social Research Council (ESRC). The ESRC had no role in the design, recruitment, analysis or preparation of this manuscript.

## Supplementary Material

Supplementary Data
