# Supplementary material for: The facilitators of communication with people with dementia in a care setting: an interview study with healthcare workers
Source: Age Ageing. 2016 Jan 13;45(1):164–70. doi: 10.1093/ageing/afv161 (PMC4711655; doi:10.1093/ageing/afv161)
Supplement: Supplementary Data [file supp_45_1_164__index.html]

Supplementary Data 

# The facilitators of communication with people with dementia in a care setting: an interview study with healthcare workers

## Supplementary Data

Supplementary Data

- Supplementary Data - Doc file
