# Supplementary material for: The facilitators of communication with people with dementia in a care setting: an interview study with healthcare workers
Source: Age Ageing. 2016 Jan 13;45(1):164–70. doi: 10.1093/ageing/afv161 (PMC4711655; doi:10.1093/ageing/afv161)
Supplement: Supplementary Data [file supp_afv161_afv161supp.doc]

**Interview Schedule**

| Main questions | Prompts and probes |
| --- | --- |
| 1. Just to start off with: In a couple of sentences, what does your job involve on a daily basis? 2. For how long have you been working with people with dementia? 3. Can you explain to me some of the challenges of communicating with people with dementia? 4. Are there any strategies that you’ve found that help them understand what you’re saying or what you want them to do? 5. Is there anything that can help you to understand them? 6. People with dementia sometimes become worried or agitated, is there anything, in your view, that can help prevent that? 7. Is there anything that hinders communication? What doesn’t work? 8. Do you think there is anything that especially characterises conversation with people with dementia? Do you think in general you speak differently to dementia patients than to a friend or colleague? 9. Have you ever had any training in communication? 10. Do you think there’s anything important that we haven’t talked about? | Has your job always been the same or has it changed over the years?  Probe 1: Strategies to encourage participation in communication.  Probe 2: Different for different residents?  Prompt: Maybe an approach or attitude?  Probe 1: Do you do these things consciously?  Probe 1: Do you think these things help with communication?  Probe 2: Do you feel you have much chance for a proper chat with the residents?  Probe 3: Do you ever share your own thoughts and feelings with the residents? Do you/they enjoy this?  Probe 1: Was this helpful? In what way? What did it involve? |
